# Dexmedetomidine sedation combined with epidural anesthesia for laparoscopy in a patient with suspected tuberculosis: A case report

**DOI:** 10.1097/MD.0000000000012144

**Published:** 2018-08-21

**Authors:** Yanming Kang, Juan Ni, Lan Wu

**Affiliations:** a West China Second University Hospital; b Key Laboratory of Birth Defects and Related Diseases of Women and Children (Sichuan University), Ministry of Education.

**Keywords:** dexmedetomidine, epidural anesthesia, nosocomial infection, sedation, tuberculosis

## Abstract

**Rationale:**

We herein present a case in which satisfactory analgesia and sedation were provided by the combination of epidural anesthesia with dexmedetomidine for exploratory laparoscopy in a patient who was highly suspected to have tuberculosis. This is an optimal anesthesia method to minimize the risk of nosocomial infection, especially in developing countries that lack sterilizers for anesthesia machines.

**Patient concerns:**

A 45-year-old woman suspected to have active tuberculosis was scheduled to undergo laparoscopy for definitive diagnosis of a pelvic mass.

**Diagnoses:**

Tuberculosis was diagnosed by exploratory laparoscopy.

**Interventions:**

The surgery was performed under general anesthesia to prevent pain and discomfort during the procedure. However, ventilation machine used by patients with tuberculosis may have a potential risk of nosocomial infection and need disinfection by a special sterilizer machine even when using a bacterial/viral filter-heat and moisture exchanger. Therefore, the surgery was performed under continuous epidural anesthesia combined with dexmedetomidine.

**Outcomes:**

The surgery was successfully completed, and pelvic tubercles were confirmed to be caseous necrotic tissue by pathologic examination. The patient began regular antituberculosis treatment after discharging from the gynecology department.

**Lessons:**

We conclude that administration of dexmedetomidine combined with epidural anesthesia can provide comfortable sedation for short laparoscopic procedures in patients with suspected tuberculosis. This technique can minimize the risk of nosocomial infection.

## 1 Introduction

Tuberculosis is the leading cause of death among bacterial diseases worldwide.^[1]^ Pulmonary or systemic dissemination may occur in patients with active disease, and this may manifest as miliary tuberculosis characterized by millet-shaped lesions on imaging tests such as ultrasound or computed tomography (CT).^[2]^ However, the diagnosis of tuberculosis is difficult in patients without characteristic laboratory and radiologic findings. Laparoscopic biopsy is a rapid and safe method with which to accurately identify pelvic tuberculosis. The surgery is usually performed under general anesthesia to prevent pain and discomfort during the procedure. Tracheal intubation can also ensure adequate ventilation and avoid aspiration under high abdominal pressure during laparoscopy. However, ventilation machines used by patients with tuberculosis may be associated with a risk of nosocomial infection. Special sterilizers for anesthesia machines are often lacking in developing countries with a high incidence of tuberculosis. We herein present a case involving a patient who was highly suspected to have tuberculosis, which was ultimately confirmed by biopsy, and in whom satisfactory analgesia and sedation for laparoscopy were provided by the combination of epidural anesthesia with dexmedetomidine (DEX).

## 2 Case report

**Figure 1:**
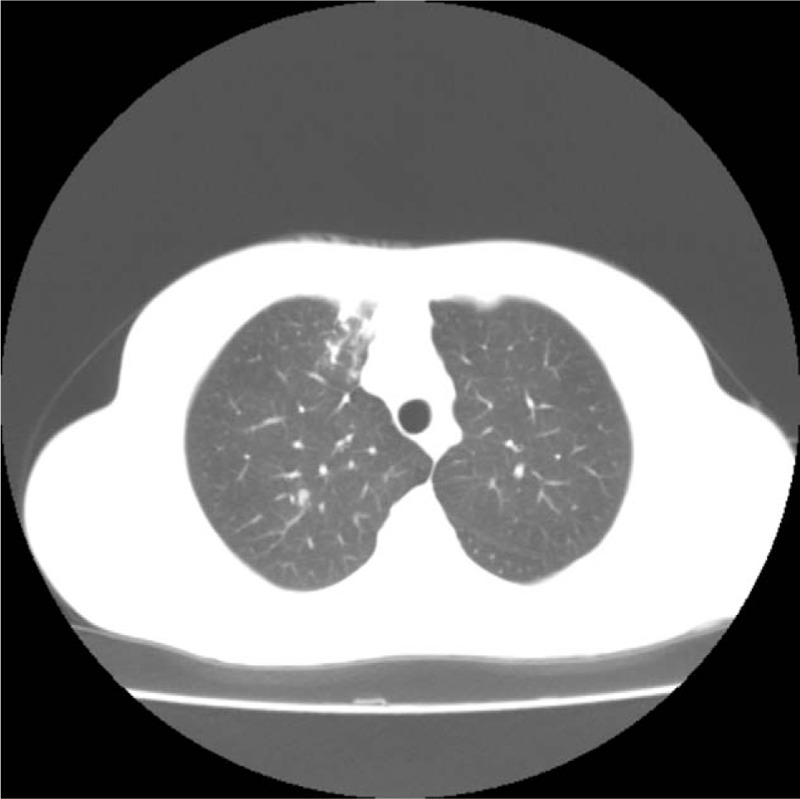
Chest computed tomography revealed evidence of infectious disease in the right upper lung lobe and enlargement of a mediastinal lymph node.

A 45-year-old woman presented to West China Hospital with a 1-month history of coughing with abdominal pain. She had no shortness of breath, hemoptysis, chest pain, night sweats, or notable marasmus. She had no significant medical history. Chest CT revealed evidence of infectious disease in the right upper lung lobe and enlargement of a mediastinal lymph node (Fig. 1). All laboratory findings were within the reference ranges with the exception of the cancer antigen 125 level, which was 482.8 U/L (reference range, 0–35 U/L). The results of a tuberculosis interferon-γ release assay and purified protein derivative test were considered positive. However, the purified protein derivative test may have a high risk of false-positive results.^[3]^ Additionally, because the sputum smear did not reveal acid-fast bacilli, tuberculosis could not be diagnosed and she did not undergo antituberculosis treatment.

**Figure 2:**
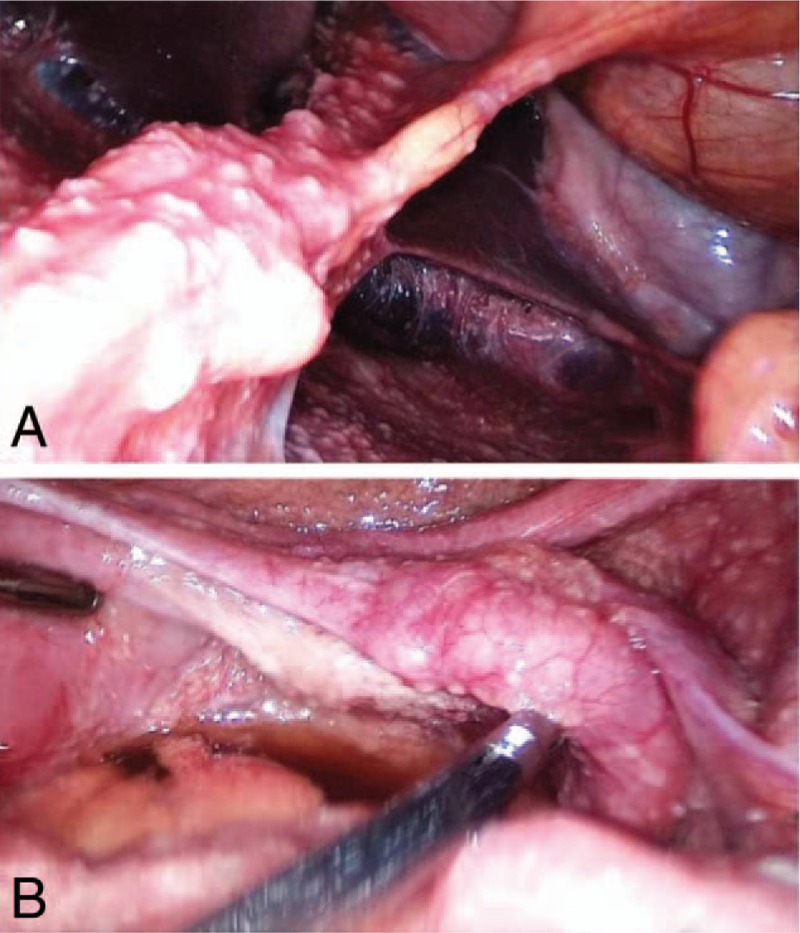
(A) Laparoscopic examination showed extensive gray tubercles located on the surface of the liver and abdominal wall. (B) Laparoscopic examination also showed yellowish peritoneal fluid and extensive gray tubercles located on the surface of the right ovary and fallopian tube.

**Figure 3:**
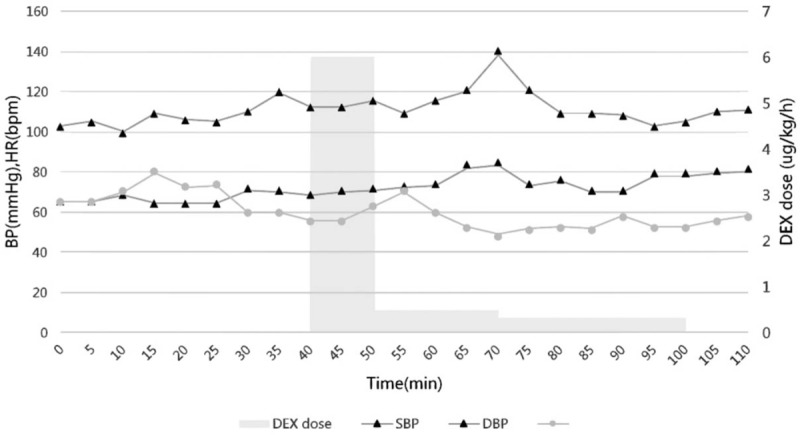
Vital signs and dexmedetomidine doses. bpm = beats per minute, DBP = diastolic blood pressure, DEX = dexmedetomidine, HR = heart rate, SBP = systolic blood pressure.

One month later, the patient was admitted to our hospital for abdominal pain. No abnormalities were found in a physical gynecologic examination. B-scan ultrasound showed a 3.0- × 1.7- × 2.2-cm right adnexal cystic mass and a 1.5- × 1.3- × 1.2-cm plaque with weak echogenicity. Abdominopelvic CT demonstrated multiple solid-cystic nodules located on the surface of the bilateral adnexa, a small amount of abdominal effusion, and multiple nodules in the thickened omentum, fascia, and peritoneum. The pelvic mass was suspected to be either tuberculosis or ovarian cancer. Therefore, the patient was scheduled to undergo exploratory laparoscopy for a definitive diagnosis. Lumbar CT showed bulging of the lumbar discs at L3-4 and L4-5 without spinal tuberculosis or a cold abscess. After standard monitoring according to the American Society of Anesthesiologists guidelines, the patient was placed in the lateral decubitus position, and epidural anesthesia was established in one attempt by insertion of an 18-gauge Tuohy epidural needle into the L1-2 space. The epidural catheter was inserted smoothly. Next, 3 mL of 2% lidocaine was injected as a test dose. Five minutes later, 6 mL of 2% lidocaine was given via the epidural catheter. DEX was continuously infused by an intravenous pump with different speed capabilities. In the first 10 minutes, the total dosage was 1 μg/kg. The speed was thereafter maintained at 0.3 to 0.5 μg/kg per hour. After administration of a loading dose of DEX, 5 mL of 2% lidocaine was incrementally given via the epidural catheter to obtain a T8 sensory level. The DEX was adjusted to keep the Ramsay sedation score (RSS) at 3 to 4 during surgery. One 10-mm optic trocar was inserted above the umbilicus in the Trendelenburg position, and 2 working trocars were inserted through the left inferolateral abdominal wall. The carbon dioxide pneumoperitoneum pressure was maintained at 11 mm Hg. The patient complained of discomfort during exploration of the upper abdomen; her discomfort was resolved by administration of 1 mg of midazolam. Laparoscopic examination showed extensive gray tubercles on the surface of the liver, omentum, intestinal canal, abdominal wall, and internal genitalia (Fig. 2A and B). Adhesions and yellowish peritoneal fluid were present in the abdominopelvic cavity. During the operation, the patient's vital signs were stable and no hypotension was observed. The surgical operation lasted for 35 minutes, and her vital signs are shown in Figure 3. The RSS recovered to 2 approximately 15 minutes after stopping the DEX administration. No adverse effects, including psychological conditions, were observed during the surgery or 1-day follow-up. The pelvic tubercles were confirmed to be caseous necrotic tissue by pathologic examination.

## 3 Discussion

The patient in the present case was suspected to have tuberculosis, which could not be distinguished from a gynecologic tumor. Because the treatment and prognosis of these 2 diseases are totally different, accurate diagnosis is particularly important. Laparoscopy is an effective method with which to distinguish pelvic tuberculosis from ovarian carcinoma. When choosing the method of anesthesia, the risk of nosocomial transmission of tuberculosis and the comfort of the patient should be considered.

Tuberculosis is spread via airborne particles measuring 1 to 5 μm and can be suspended in air for several hours.^[4]^ Furthermore, mycobacterial tuberculosis can withstand weak disinfectants and can survive in a dry state for several weeks. Mycobacterial tuberculosis microorganisms are retained and propagate within the ventilatory machine, and thorough disinfection requires a special sterilizer machine. Although domestic filters are used, there are no specifications regarding filtering of mycobacterium tuberculosis. Sharing the same anesthetic circuit increases the risk of patient-to-patient transmission of tuberculosis. Moreover, health care workers, especially anesthetists, are at constant risk of acquiring tuberculosis by inhalation of droplet nuclei during airway manipulation.^[4,5]^ The maneuvers for intubation, suctioning, or tracheal extubation may lead to intensive contact with bronchial secretions and will markedly increase the duration of exposure and risk of infection. Teo et al^[4]^ and Freytag et al^[6]^ reported cases in which anesthetists were suspected to have been infected by a patient with tuberculosis while performing general anesthesia. To avoid nosocomial infection in the present case, we used a one-off oxygen inhalation device during the operation under epidural anesthesia. A sterilized simple respirator was kept on standby if needed for total spinal anesthesia. In addition to the risk of infection, general anesthesia may also have a potential safety hazard. Wang et al^[7]^ and Chung et al^[8]^ described 2 cases of hemoptysis induced by positive-pressure ventilation during general anesthesia. This is an unusual but often lethal complication. Therefore, epidural anesthesia is superior to general anesthesia for such patients.

In the present case, we chose continuous epidural anesthesia at L1-2 to attain a sufficient blocked level. High intra-abdominal pressure and the Trendelenburg position cause brain swelling and shortness of breath. Therefore, we recommend performance of the operation by a skilled surgeon as well as reductions in the abdominal pressure and angle of the Trendelenburg position. Proper sedation can relieve the patient's anxiety and discomfort. If the frozen section biopsy confirms an ovarian tumor and further surgical treatment is required, the epidural anesthesia can also be used for removal of a benign tumor or the catheter can be used for postoperative analgesia. If the biopsy indicates that the tumor is malignant, general anesthesia can be applied without fear of hospital infection.

In contrast to traditional sedatives such as midazolam or propofol, DEX has the obvious advantage of mirroring natural sleep without obvious circulatory or respiratory depression. DEX is a selective α2-adrenoreceptor agonist^[9]^ and has beneficial sedative, anxiolytic, and analgesic properties. In the present case, the RSS was maintained at 3 to 4. The patient woke up at the beginning of surgery but felt no pain or anxiety. Kunisawa et al^[10]^ reported a case in which DEX was safely administered for sedation during spinal anesthesia in a very old patient, and the respiratory and circulatory parameters remained relatively stable. Our patient also remained hemodynamically stable. The blood pressure exhibited dimorphic changes. Administration of a high dose of DEX in the early stage can directly stimulate vascular smooth muscle α2-receptors to produce a hypertensive response.^[11]^ In the present case, the basal blood pressure of 103/65 mm Hg gradually increased until reaching the highest intraoperative blood pressure of 138/83 mm Hg. DEX can cause a temporary reduction of blood pressure by anti-sympathetic nerve excitation.^[11]^ With the adjuvant effect of midazolam, the blood pressure returned to baseline levels. Our patient's heart rate decreased from 60 to 49 bpm by the dual effect of the initial dose of DEX and the carbon dioxide pneumoperitoneum. However, cardiovascular drug administration was not required at this time because the decrease in the heart rate was not critical. Patients who receive DEX require strict dynamic observation and close monitoring. Our patient also maintained spontaneous respiration and airway patency. DEX is unlikely to cause respiratory suppression such as that seen with propofol. The airway obstruction caused by propofol may augment abdominal breathing, thereby possibly impacting the operation.

In conclusion, administration of DEX combined with epidural anesthesia can provide comfortable sedation for short laparoscopic procedures in patients with suspected tuberculosis. Epidural anesthesia can reduce the risk of tuberculosis transmission and has advantages in terms of postoperative analgesia. Patients with contraindications to epidural or intraspinal anesthesia should undergo general anesthesia. In such cases, however, adequate personnel protection should include highly efficient facemasks, bacterial/viral filters, and disinfection of the anesthesia machine.

## 4 Consent

Written informed consent was obtained from the patient for publication of this case report and any accompanying images. A copy of the written consent is available for review by the Editor of this journal.

## Author contributions

**Conceptualization:** Yanming Kang, Lan Wu.

**Data curation:** Yanming Kang.

**Resources:** Yanming Kang.

**Writing – original draft:** Yanming Kang.

**Writing – review & editing:** Lan Wu, Juan Ni.
